# Co-designed interventions package for enhancing women's resilience in reproductive and child health through income-generating associations: a human-centered design study in Tanzania

**DOI:** 10.3389/frhs.2026.1856655

**Published:** 2026-06-25

**Authors:** Kahabi Ganka Isangula, Aminieli Itaeli Usiri, Eunice Siaity Pallangyo

**Affiliations:** School of Nursing and Midwifery, Aga Khan University, Dar Es Salaam, Tanzania

**Keywords:** human centered design, income generating associations, maternal and child health, maternal and child mortality, sub-Saharan Africa, Tanzania

## Abstract

**Background:**

Maternal and under-five mortality remain major public health concerns in many low- and middle-income countries, including Tanzania. Despite their widespread use in strengthening economic resilience, income-generating associations (IGAs) have not fully utilized their potential as platforms for addressing reproductive and child health (RCH) challenges.

**Objective:**

This study aimed to co-design an intervention package with women participating in income-generating associations to enhance their preparedness and resilience in addressing RCH challenges in Tanzania.

**Methods:**

A qualitative descriptive study guided by a Human-Centered Design (HCD) framework was conducted in the Arusha and Shinyanga regions of Tanzania. The study followed four iterative phases: (1) discovery and mapping of IGAs to understand members’ experiences and RCH challenges; (2) co-design and prototyping of potential interventions with women and stakeholders; (3) validation of the prototype intervention package with additional IGA members; and (4) refinement of the package through consultations with reproductive and child health experts. Data was collected through focus group discussions, key informant interviews, and consultative workshops with individuals participating in IGAs, community development officers, IGA coordinators, and RCH stakeholders. Qualitative data were analyzed using thematic analysis, while intervention priorities were ranked based on acceptability, feasibility, and sustainability.

**Results:**

The discovery phase revealed that IGAs function as important economic resilience mechanisms for women but are rarely utilized as platforms for reproductive and child health promotion. Key barriers included limited health education within groups, financial constraints affecting access to healthcare, gender norms influencing participation, and limited engagement among men. Through the co-design process, several candidate interventions were generated and prioritized. Four core components emerged as the most feasible and acceptable: (1) delivery of maternal and child health education through regular IGA meetings; (2) facilitation of health insurance awareness and enrollment among members; (3) strengthening governance and coordination within IGA groups; and (4) increasing participation and support for vulnerable women, including adolescent girls and young women. Validation with additional participants confirmed the feasibility and relevance of these interventions within the existing IGA structure.

**Conclusion:**

The co-designed intervention package emphasizes the necessity of integrating health education, financial protection mechanisms, and governance strengthening within existing economic groups.

## 1 Introduction

Despite substantial global progress in reproductive and child health (RCH), maternal and child mortality remain major public health concerns, particularly in low- and middle-income countries (LMICs). The World Health Organization (WHO) estimated that approximately 287,000 women died from pregnancy-related causes in 2020, indicating only modest improvement since the adoption of the Sustainable Development Goals (SDGs) in 2016 (1). Similarly, the United Nations Children's Fund (UNICEF) reports that global under-five mortality declined by 59% between 1990 and 2021, from 93 to 38 deaths per 1,000 live births (2). Despite this progress, reductions have been uneven across regions, with the burden of maternal and child mortality remaining disproportionately high in low-income settings.

Tanzania has made notable strides in improving RCH and maternal and child health (MCH) outcomes in recent decades. However, maternal mortality and under-five mortality remain important public health concerns. According to the Tanzania Demographic and Health Surveys (TDHS), maternal mortality ratios have shown fluctuations between 2015 and 2022, reflecting both periods of progress and setbacks (3). While the under-five mortality rate has declined, it remains above desired global targets (3). These outcomes are influenced by a complex interplay of structural, community, and individual factors. At the community level, poverty, harmful cultural beliefs, limited health literacy, and inadequate social support systems often constrain women's access to and utilization of RCH services (4, 5). For example, low household income can limit the ability to seek timely care, while cultural norms and limited awareness about maternal and child health services contribute to delayed or inadequate care-seeking behaviors (6–8).

In response, the Government of Tanzania and its partners have implemented several interventions aimed at improving maternal and child health outcomes. These initiatives include strengthening health systems, expanding access to essential medicines and supplies, improving health facility infrastructure, and enhancing the capacity of healthcare workers (9–11). Additionally, innovative programs such as community health worker initiatives and digital health interventions including the *m-Mama* emergency transport system implemented in partnership with Vodacom, have been introduced to improve access to maternal and neonatal services (12, 13). While these interventions have contributed to progress, persistent disparities suggest that additional community-responsive strategies are required.

The WHO emphasizes community engagement as a critical component of effective RCH interventions (14, 15). However, many health programs in Tanzania and other LMICs continue to be designed with limited involvement of the communities they intend to serve. This lack of participation can result in interventions that do not fully reflect local priorities, social dynamics, or contextual realities, ultimately limiting their effectiveness and sustainability (16–19).

Women play a central role in maintaining family health and wellbeing, making RCH issues directly relevant to their lived experiences. Nevertheless, in many LMICs, including Tanzania, women often have limited decision-making power over financial resources, mobility, and healthcare choices, which can hinder timely access to essential maternal and child health services (20, 21). Structural gender inequalities, poverty, and dependence on male partners for financial support frequently constrain women's ability to seek appropriate reproductive and child healthcare, particularly in rural and resource-limited settings. These challenges highlight the importance of interventions that simultaneously strengthen women's economic resilience, social support systems, and health-related decision-making capacity.

Recent socioeconomic shifts across Sub-Saharan Africa have promoted women's economic empowerment through participation in community-based financial and social associations. In Tanzania, income-generating associations (IGAs) have emerged as important grassroots mechanisms for enhancing women's economic independence, collective solidarity, and social networking opportunities (22). These IGAs encompass a broad range of community-based structures, including Village Community Banks (VICOBA), women's self-help groups, rotating savings and credit associations, farmers’ groups, and other informal savings and lending networks. Although these groups differ in organizational structure and operational approaches, they share common goals related to economic empowerment, mutual support, savings mobilization, and livelihood strengthening among members.

Many of IGAs are initiated, organized, and managed by women themselves, creating socially trusted spaces for peer interaction, knowledge exchange, and collective problem-solving. Beyond their economic functions, IGAs provide opportunities for savings, micro-credit access, entrepreneurship development, and social cohesion, all of which may indirectly influence health-seeking behaviors and household decision-making processes (23, 24). Through regular meetings and strong interpersonal networks, these groups may also serve as practical community platforms for delivering health education, promoting financial preparedness for maternal and child health needs, facilitating health insurance awareness, and strengthening women's collective capacity to respond to reproductive and child health challenges. Despite this potential, the integration of reproductive and child health interventions within existing IGAs remains relatively underexplored in many LMIC settings, including Tanzania.

Recognizing this opportunity, the Aga Khan University School of Nursing and Midwifery implemented a Human-Centered Design (HCD) study to collaboratively develop interventions aimed at strengthening women's resilience in addressing RCH challenges through IGAs. The novelty of this study lies in leveraging existing women's economic associations as integrated platforms for health promotion and resilience-building, rather than establishing parallel health-specific groups or externally driven community structures. Building on already established social and economic networks seeks to enhance the feasibility, acceptability, and sustainability of RCH interventions within women's everyday livelihood contexts. HCD is a participatory and user-centered problem-solving approach that places end-users at the center of the innovation process, ensuring that solutions are grounded in the lived experiences, priorities, and contextual realities of the communities they are intended to serve (25, 26). Unlike the traditional top-down intervention development approaches, HCD emphasizes continuous engagement with stakeholders throughout the design process, enabling researchers and communities to jointly identify problems, explore potential solutions, and refine interventions based on iterative feedback (25). Through iterative engagement, empathy-driven inquiry, co-creation, rapid prototyping, and continuous refinement, HCD enables stakeholders to develop interventions that are contextually appropriate, acceptable, feasible, and potentially sustainable within local settings (25). In public health and maternal and child health research, HCD has increasingly been recognized as an important approach for improving community ownership, intervention relevance, and implementation success, particularly in complex and resource-constrained environments where social, cultural, and economic factors strongly shape health behaviors and service utilization (26).

Therefore, this study employed an HCD approach to co-design an intervention package with women participating in IGAs and key stakeholders, with the goal of enhancing women's resilience, knowledge, decision-making capacity, and collective engagement in addressing reproductive and child health challenges. The resulting intervention package is intended to strengthen women's economic and social empowerment while improving their capacity to respond to maternal and child health needs. If found feasible and acceptable, this intervention package could provide a scalable strategy for strengthening community-driven RCH initiatives in Tanzania and similar low-resource settings.

## 2 Methods

### 2.1 Design

A detailed protocol for this study has been published previously (27). Briefly, this study employed a qualitative descriptive design within a HCD framework to co-develop interventions aimed at strengthening women's resilience in addressing RCH challenges. The qualitative approach was selected to enable an in-depth exploration of women's experiences, perceptions, and contextual realities within VICOBAs. The use of HCD was particularly relevant for this study because existing maternal and child health interventions in many low-resource settings are often developed using top-down approaches with limited engagement of end-users, potentially reducing contextual relevance, acceptability, and long-term sustainability (26). The HCD framework therefore provided an opportunity to address important empirical and contextual gaps by ensuring that women participating in IGAs, community leaders, and reproductive health stakeholders actively contributed to identifying challenges, generating solutions, and refining intervention priorities.

The HCD approach was implemented through an adaptation of the traditional five-step model into four iterative and interconnected phases tailored to the sociocultural and operational context of community-based income-generating associations in Tanzania (26). The adapted steps included (1) discovery and inquiry, (2) consultative co-design and prototyping, (3) validation and insight gathering, and (4) refinement and adaptation. The iterative nature of these phases allowed findings from one stage to directly inform the next stage, enabling continuous learning, reflection, and refinement of proposed interventions based on stakeholder feedback and contextual realities. The framework was adapted to fit the local sociocultural and operational context of Tanzanian IGAs by incorporating community dialogue sessions, participatory scoring processes, and stakeholder validation workshops involving women, community development officers, IGA coordinators, and reproductive and child health experts. This adaptation was intended to enhance contextual responsiveness of the emerging intervention package in addressing local priorities, strengthen stakeholder participation, existing group structures, implementation realities within community-based economic associations and ensure continuous refinement of intervention ideas throughout the co-design process.

The co-design process addressed three guiding components:.

1. Design problem: How women participating in IGAs (e.g., VICOBA groups) can strengthen their resilience and readiness to address reproductive and child health challenges.
2. Research question: What barriers limit the effectiveness of IGAs in supporting women to address RCH challenges?
3. Design question: What feasible, acceptable, and sustainable intervention package can be co-developed with women and stakeholders to address these barriers?

This design allowed for a comprehensive understanding of the social, cultural, economic, and gender-related contexts shaping women's experiences while facilitating the development of a contextually grounded and stakeholder-informed intervention package.

### 2.2 Settings

The study was conducted in the Shinyanga and Arusha regions of Tanzania, selected to capture diverse sociocultural and geographic contexts. Shinyanga Region, located in northwestern Tanzania and historically part of the Sukuma cultural area, is predominantly rural and characterized by relatively poor reproductive and child health indicators (28). Women in this setting often experience limited decision-making autonomy and rely on multiple healthcare systems, including traditional and biomedical services. Shinyanga Municipal Council was specifically selected due to the presence of numerous active IGAs and existing community networks supported by SONAM. Arusha Region, located in northern Tanzania, represents a more ethnically diverse context that includes communities such as the Maasai, where patriarchal social structures often influence household decision-making. Within this region, Arumeru District was selected because it includes both rural and peri-urban communities, allowing for the exploration of varied experiences of women participating in IGAs.

### 2.3 Study population, sample size, and sampling

The study involved women participating in income-generating associations, IGA coordinators, community development officers, and reproductive and child health stakeholders.

### 2.4 Step 1: discovery inquiry

The first phase involved a qualitative collection to identify barriers that limit the ability of IGAs to strengthen women's resilience in addressing RCH challenges. A total of six focus group discussions (FGDs) were conducted (three in each region), each involving 6–8 women and men participating in IGAs. Additionally, ten key informant interviews (KIIs) were conducted with municipal IGA coordinators and community development officers (five in each region). Participants were purposively selected with support from IGA coordinators and community development officers. Interviews were conducted at locations convenient to participants. Prior to data collection, research assistants explained the study objectives and obtained written informed consent. Each discussion lasted approximately 45–60 min and was audio-recorded with participants’ permission. The collected data were transcribed, managed, and analyzed using thematic analysis (29) with Nvivo Software Version 12 (Lumivero, formerly QSR International), generating insights that informed the subsequent co-design phase.

### 2.5 Step 2: consultative co-design meetings

In the second phase, a two-day co-design workshop was conducted to collaboratively develop a prototype intervention package. A transdisciplinary group of 30 participants was convened, including 15 women from IGAs, 5 IGA coordinators, 5 community development officers, and 5 stakeholders from organizations involved in RCH programs. The workshop included three key sessions:

1. *Synthesis session:* Participants reviewed and validated findings from the discovery phase, sharing additional perspectives on challenges affecting women in IGAs.
2. *Ideation session:* Participants brainstormed potential solutions to the identified challenges.
3. *Prototyping and co-creation*: Participants developed preliminary intervention models and discussed implementation components such as delivery mechanisms and responsible actors.

During the co-design workshops, proposed solutions were evaluated collaboratively using predefined prioritization criteria, including acceptability, feasibility, perceived impact, and sustainability. Participants worked in small groups to rate each proposed intervention on a scale ranging from 0 to 10, with higher scores indicating greater perceived suitability and priority for implementation. Rather than conducting independent individual scoring, the process emphasized collective reflection and participatory decision-making, consistent with HCD principles.

Following the initial scoring exercise, facilitator-led discussions were conducted to review variations and discrepancies in group ratings, encourage participants to explain the rationale behind their scores, and promote dialogue around differing perspectives. Through these moderated discussions, participants collectively refined their assessments and generated consensus-based cumulative scores for each intervention option. Scores from all groups were subsequently compiled and summarized using Microsoft Excel to support comparison and ranking of proposed solutions. The highest-ranked and most contextually relevant solutions informed the development of the initial intervention prototype. A sample scoring sheet used during the prioritization exercise has been provided as Supplementary Material to enhance methodological transparency and reproducibility.

### 2.6 Step 3: validation and insight-gathering inquiry

The third phase aimed to validate and refine the prototype intervention package by engaging women in IGAs who had not participated in earlier phases. A total of six additional FGDs (three per region) were conducted with women from different communities within the selected districts. Participants were recruited with assistance from community development officers. Participants reviewed the prototype components and evaluated them using the same criteria: *acceptability, feasibility,* and *sustainability*, on a 0–10 rating scale. Qualitative data from these discussions were managed and analyzed using thematic analysis (29) in Nvivo Software Version 12 (Lumivero, formerly QSR International), while quantitative ratings were analyzed using Microsoft Excel. The feedback obtained during this phase informed revisions to the intervention prototype.

### 2.7 Step 4: refinement and adaptation workshop

The final phase involved a one-day consultative workshop with approximately 12 reproductive and child health stakeholders, including professionals from Aga Khan University and representatives from the Tanzania Midwifery Association. Participants reviewed the results from the validation phase and worked collaboratively to refine the intervention package. The goal was to produce a finalized intervention package suitable for future pilot testing and evaluation, pending the availability of external funding.

### 2.8 Data management and analysis

Data collection was conducted by two trained research assistants at each study site, both holding diplomas in medicine and community development. Prior to data collection, the research assistants received orientation on the study objectives, qualitative interviewing techniques, ethical considerations, and procedures for maintaining confidentiality and data quality. The assistants were responsible for facilitating interviews and discussions, transcription of audio recordings, and translation of transcripts from Swahili into English. The Principal Investigator (PI) and Co-Principal Investigators (Co-PIs) supervised the overall data collection process and independently reviewed selected transcripts to verify transcription accuracy, consistency of translations, and completeness of the data.

To ensure confidentiality and anonymity, all participants were assigned pseudonyms, and identifying information was removed from transcripts before analysis. As noted above, qualitative data analysis followed a thematic analysis approach as described by Braun and Clarke (29). The analysis adopted a primarily deductive thematic analytical framework guided by the study objectives, research questions, and key domains emerging from the HCD approach. Initially, members of the research team familiarized themselves with the transcripts through repeated reading and discussion of the data. The team then collaboratively developed an analytical framework outlining preliminary themes, sub-themes, and coding categories relevant to women's resilience, reproductive and child health challenges, and the role of income-generating associations.

Transcripts were subsequently imported into NVivo Version 12 software to support systematic coding, organization, and retrieval of qualitative data. Coding was conducted iteratively using a deductive thematic analysis approach, while still allowing flexibility for the emergence of contextually relevant insights during the coding process. The research team regularly reviewed and compared coded segments to ensure consistency and alignment with the analytical framework. Codes were refined, merged, expanded, or removed through team discussions to improve conceptual clarity and thematic coherence. Codes that did not meaningfully contribute to the study objectives or broader thematic interpretation were excluded from the final analytical framework. Following coding refinement, related codes were grouped into broader themes and sub-themes to facilitate interpretation and synthesis of findings across study phases. Finally, coded outputs and thematic summaries were exported to Microsoft Word to support deeper narrative interpretation, triangulation of findings across participant groups, and development of the final intervention framework.

### 2.9 Ethics statement

This study was reviewed and approved by the Aga Khan University Research Ethics Committee (AKU/2024/02/fb/09/135). Additional permission to conduct the study was obtained from the President's Office—Regional Administration and Local Government (AB.307/323/01) and the relevant Regional and District Medical Authorities in Arusha and Shinyanga regions, Tanzania. All participants were informed about the purpose of the study, their rights as participants, and the voluntary nature of participation. Written informed consent was obtained from all participants prior to data collection. Confidentiality and anonymity were maintained throughout the study.

## 3 Results

### 3.1 Participants demographics

This study included a total of 166 participants from the Arusha (*n* = 84) and Shinyanga (*n* = 82) regions of Tanzania who participated across the four phases of the adapted HCD approach. During the discovery phase, six FGDs were conducted (three per region), involving a total of 48 participants drawn from IGAs. Of these, 25 participants were from Arusha and 23 from Shinyanga, with each FGD comprising 7–8 participants. In addition, 10 KIIs were conducted with community development officers and IGA coordinators, including five participants from each region.

The consultative co-design phase involved 60 participants (30 per region), including women and men participating in IGAs, IGA coordinators, community development officers, and RCH stakeholders. The validation phase included six additional FGDs (three per region) conducted with new IGA members who had not participated in earlier phases. Each FGD comprised approximately eight participants, resulting in a total of 48 participants. Finally, the refinement and adaptation phase engaged 12 reproductive, maternal, and child health experts who were not part of the initial community participant sample.

Most participants across all phases were women (approximately 88%), reflecting the predominantly female composition of IGAs and RCH coordination in the study settings. Male participants constituted approximately 10% of participants in the discovery phase (*n* = 6), 13% in the co-design phase (*n* = 8), 8% in the validation phase (*n* = 6), and 17% in the refinement and adaptation phase (*n* = 2). Male participants were intentionally included to provide insights into gender norms, household decision-making dynamics, and barriers affecting male engagement and support for VICOBA activities. FGDs included both male and female participants in mixed-group discussions; however, all sessions were facilitated by experienced qualitative researchers who ensured equitable participation, minimized power imbalances, and created opportunities for all participants to contribute meaningfully despite the smaller proportion of male participants.

### 3.2 Findings from discovery inquiry

Participants consistently described VICOBA as an important mechanism for strengthening women's economic resilience and improving household coping capacity. Women emphasized that participation in VICOBA enabled them to access small loans, stabilize businesses, support children's education, and respond to emergencies more rapidly than would otherwise be possible. Beyond economic benefits, the findings suggest that VICOBA functions as a socially trusted support system that promotes solidarity, mutual assistance, and collective problem-solving among women (Table 1).

**Table 1:** Discovery inquiry themes and subthemes.

Theme	Subthemes	What it means in this study
VICOBA as a pathway to women’s economic resilience	VICOBA as economic resilience infrastructure	Women described VICOBA as improving ability to pay school fees, build assets, stabilize businesses, and respond to emergencies via mutual aid/rapid access to cash/loans
Constraints limiting the use of VICOBA/IGAs as platforms for RCH/MCH	Time, poverty and role strain	Participation in VICOBA/IGAs can compete with caregiving time; “money-seeking” pressures may reduce time for family needs
	Underutilized health platform, but high potential	Participants framed IGAs as an efficient platform for health workers to deliver RCH education because members are organized and reachable
	Gender norms and low male engagement constrain IGA health leverage	Men may discourage participation and associate groups with “behavior change”/reduced “respect,” triggering household conflict and limiting health-focused collective action
	VICOBA RCH/MCH support is narrow (often limited to gifts), with demand for structured RCH/MCH funds/by-laws	Women noted limited group-level provisions for pregnancy/childbirth beyond small gifts; suggested formal RCH/MCH funds or rules to support pregnant/postpartum members
	Health insurance: low literacy/coverage and desire for group facilitation	Women highlight lack of insurance understanding and affordability; interest in group insurance facilitation and education

Most women explained that the ability to rapidly mobilize financial support during illness, bereavement, or family emergencies reduced dependence on external assistance and enhanced their sense of financial autonomy. In this context, VICOBA was not only perceived as an economic structure but also as a social resilience mechanism capable of supporting women during periods of vulnerability.

“(VICOBA) has helped me: I have educated my children through secondary school… they have even helped my business… they have helped me in many ways, truly.” (FGD Participant, Arusha)

“If someone falls suddenly ill… we call the leaders… they withdraw money… if it is urgent we borrow from a member, then repay once we withdraw from the bank.” (FGD Participant, Shinyanga)

These findings reinforce the growing recognition that community-based financial groups can contribute not only to women's economic empowerment but also to broader social resilience and collective wellbeing. Importantly, the organizational structure and regular interactions within IGAs appeared to create opportunities for integrating additional social and health-related interventions.

#### 3.2.1 Constraints limiting the use of VICOBA/IGAs as platforms for Rch/Mch resilience

Despite the recognized economic value of VICOBA, participants overwhelmingly reported that these groups were not currently optimized to support reproductive and child health outcomes. The barriers identified operated across multiple levels including individual, interpersonal, organizational, and structural domains, reflecting the complex realities shaping women's participation and health decision-making.

At the individual and household levels, women described tensions between economic survival activities and caregiving responsibilities. Although participation in IGAs improved financial opportunities, some women explained that pressures associated with income generation could reduce time available for childcare and family support.

“I think many mothers participating in VICOBA… don’t have time for their families… they are chasing money… they may leave in the morning and return at night…” (FGD participant, Shinyanga)

At the organizational level, participants repeatedly emphasized that IGAs represented an underutilized platform for health promotion. Women and stakeholders noted that VICOBA groups already possess important characteristics required for community health interventions, including regular meetings, social trust, organized leadership structures, and accessibility to women within communities. However, these existing structures had rarely been leveraged for structured maternal and child health education or reproductive health promotion.

 “It’s a very good platform… it can be used by health professionals to reach women and provide education, but it is currently not used for this…” (KII Participant, Arusha)

Gender norms and household power dynamics also emerged as important structural barriers. Participants explained that some men perceived VICOBA participation negatively, associating women's involvement with reduced “respect,” behavioral change, or threats to traditional household authority. These perceptions sometimes contributed to household conflict and discouraged women's participation in both economic and health-related group activities.Furthermore, the framing of VICOBA as primarily targeting women at its inception created the perception that it is a “women’s activity,” thereby limiting male participation.

“Some men say groups are harmful… after the wife joins, she ‘develops bad behaviors’… the husband fights for her not to attend…” (KII Participant, Arusha)

These findings suggest that efforts to integrate RCH interventions within IGAs may require not only women-focused strategies but also broader household and community engagement approaches addressing gender relations and social perceptions.

Participants further highlighted important gaps in structured maternal and child health support within VICOBA groups. Existing support mechanisms were often informal and limited to small gifts or temporary assistance following childbirth. Women therefore proposed establishing formalized maternal and child health support funds and group by-laws to improve preparedness for pregnancy, childbirth, and maternal emergencies.

Another prominent finding involved widespread concerns regarding health insurance access and affordability. Women described both informational and financial barriers related to health insurance enrollment, with many participants reporting catastrophic out-of-pocket expenditures during illness and pregnancy-related complications. Participants strongly supported the idea of integrating health insurance education and facilitation into existing IGA activities. “I have continued to suffer … I’ve been admitted to (tertiary hospital) many times without insurance … I really wished for insurance … VICOBA need to facilitate insurance for its members and education on insurance packages” (FGD participant, Arusha).

Collectively, the discovery inquiry findings demonstrate that while IGAs already function as important economic resilience structures, they remain underutilized as platforms for strengthening reproductive and child health resilience. The findings also highlight the interconnected influence of financial vulnerability, gender norms, health literacy, and social organization on women's maternal and child health experiences. These insights directly informed the subsequent co-design and prioritization of intervention components.

### 3.3 Findings from consultative co-design meetings

The consultative co-design phase built upon findings from the discovery inquiry by engaging participants in collaborative reflection, solution generation, prioritization, and prototype development. The findings from this phase demonstrate how participants moved beyond identifying barriers to actively co-creating practical and contextually grounded solutions for strengthening RCH/MCH resilience within IGAs.

#### 3.3.1 Validation and expansion of discovery findings

During the synthesis sessions, participants broadly agreed that the findings generated during the discovery inquiry accurately reflected the realities experienced by women participating in VICOBA and other IGAs. Participants further expanded discussions around gender dynamics and low male engagement, providing deeper insight into the social and cultural factors influencing participation in group-based activities.

Most participants explained that men often perceived VICOBA as “women’s spaces,” while others associated participation with gossip, unnecessary meetings, or threats to traditional gender roles and household authority. Male participants additionally expressed concerns that joining VICOBA might interfere with income-generating responsibilities or result in loss of leadership control within groups predominantly composed of women. These findings suggest that gender norms and perceptions of masculinity continue to shape participation in community economic and health-related initiatives. At the same time, some male participants acknowledged that increased understanding of the potential health and economic benefits of VICOBA could improve their willingness to engage or support women's participation.

“Because of time, we often can’t participate… my wife is the one in the group… I just give her my money for contributions… but this education has helped me see that I can also sit there and engage.” (Male Participant, Shinyanga)

Participants also emphasized that limited communication between women and their partners regarding VICOBA activities sometimes contributed to misunderstanding, mistrust, and reduced male support. These findings reinforced the importance of integrating household sensitization and male engagement strategies into the proposed intervention package.

#### 3.3.2 Generation of intervention ideas during ideation sessions

The ideation sessions generated a broad range of intervention ideas designed to strengthen the contribution of IGAs to maternal and child health resilience. Participants generated 34 proposed interventions in Arusha and 42 in Shinyanga. These ideas were subsequently organized into thematic solution areas including: (i) maternal and child health education through VICOBA meetings, (ii) male sensitization and engagement strategies, (iii) establishment of maternal and child health support funds and by-laws, (iv) health insurance education and facilitation, and (v) strategies to strengthen participation of adolescent girls and young women (AGYW).

Importantly, many proposed interventions sought to leverage existing organizational structures within IGAs rather than introducing entirely new community systems. This reflects one of the central strengths of the HCD approach employed in this study: participants prioritized interventions that were perceived as realistic, culturally acceptable, and operationally feasible within existing community contexts.

Participants proposed that maternal and child health education could be delivered during routine VICOBA meetings through collaboration with healthcare workers, distribution of educational materials, and integration of dedicated health sessions into regular group agendas. This proposal reflected participants’ recognition that existing social networks and meeting structures could serve as efficient and sustainable entry points for health promotion activities.

Similarly, participants proposed that male engagement could be strengthened through community sensitization campaigns and mobilization of existing male VICOBA members as peer advocates. This recommendation reflects recognition that sustainable improvement in reproductive and child health outcomes requires broader household and community support beyond women alone.

The establishment of structured maternal and child health support mechanisms also emerged as an important priority. Participants recommended creating dedicated maternal and child health funds within VICOBA groups, supported through small member contributions and coordinated through formal group governance structures. This proposal demonstrates how participants linked financial resilience with maternal health preparedness and emergency response.

Another major area of concern involved financial protection and healthcare affordability. Participants strongly supported integrating health insurance education and facilitation into VICOBA activities. Women viewed IGAs as potentially effective platforms for increasing awareness of insurance schemes, supporting group-based enrollment mechanisms, and strengthening collective saving for healthcare needs.

Finally, participants emphasized the importance of strengthening inclusion of AGYW, particularly those from economically vulnerable households. Proposed strategies included mentorship models linking younger women with experienced members, targeted recruitment efforts, and facilitating participation in income-generating activities through existing VICOBA structures.

#### 3.3.3 Prioritization and development of rough prototypes

During the prototyping phase, participants collectively evaluated proposed interventions based on acceptability, feasibility, and sustainability using a 0–10 scoring scale. The scoring process combined individual ratings with facilitated consensus discussions, enabling participants to critically reflect on implementation realities and prioritize interventions perceived as most achievable within their communities.

The scoring process revealed notable similarities across regions regarding intervention priorities. In Arusha, highly ranked interventions included strengthening VICOBA governance structures, entrepreneurship education, maternal and child health education, establishment of maternal health support funds, nutrition-focused activities, and health insurance facilitation. In Shinyanga, participants agreed on a cutoff score of ≥75/90 to identify interventions suitable for inclusion within the preliminary “rough prototype.” (Table 2).

**Table 2:** Summary of key findings from co-creation phase.

Co-design output	Key findings
Synthesis: discovery findings were validated and expanded	Participants broadly agreed discovery findings reflect reality; male engagement discussion was expanded (time constraints, perceptions VICOBA is “for women,” dislike of meeting “gossip,” fear of male dominance)
Ideation: large solution space generated	Draft reports 34 ideas (Arusha) and 42 ideas (Shinyanga), grouped into categories such as health education via VICOBA, male sensitization, MCH fund/by-laws, insurance education, adolescent/young women engagement
Prototyping/selection: “rough prototype” chosen using acceptability/feasibility/sustainability ratings	Grouped intervention ideas were collaboratively rated by different stakeholder groups based on acceptability, feasibility, and potential sustainability using a 0–10 scoring scale. The cumulative score for each intervention was calculated out of a maximum of 90 points, and a predefined cutoff score of ≥75/90 was used to determine eligibility for inclusion in the rough prototype intervention package.

Following triangulation of findings across both regions, a total of nine candidate interventions were identified during the co-design process. However, only four interventions achieved the predefined cutoff threshold of ≥75/90 and were therefore retained as components of the final rough prototype (Table 3). Interventions that scored below the cutoff criteria were excluded from the final rough prototype package. The four highly prioritized intervention components included:

1. Facilitation of health insurance awareness and enrollment among VICOBA members;
2. Delivery of maternal and child health education through VICOBA meetings;
3. Strengthening governance and coordination within VICOBA groups; and

**Table 3:** Final rough prototype and other interventions from co-creation phase.

Category	Rank	Intervention	Score	Relative bar
The final rough prototype	1	Facilitate health insurance among VICOBA members	87	██████████████████████████████
	2	Maternal and child health education via VICOBA	85	█████████████████████████████
	3	Improve VICOBA governance/management	78	███████████████████████████
	4	Increase impact for vulnerable groups (AGYW/poor women)	77	███████████████████████████
Interventions that did not meet the cut-off criteria.	5	Financial/loan/relationship education	73	█████████████████████████
	6	Education for women <30 on VICOBA/entrepreneurship	73	█████████████████████████
	8	Increase male engagement	67	███████████████████████
	7	Increase use of technology to manage VICOBA	61	█████████████████████
	9	Community sensitization	47	████████████████

Increasing support for vulnerable women, including adolescent girls and young women from low-income households. The prioritization findings demonstrate that participants favored interventions that integrated economic resilience, health education, governance strengthening, and social inclusion. These findings also illustrate the practical value of HCD in generating interventions that are simultaneously community-driven, contextually relevant, and implementation-oriented.

Notably, some interventions such as community sensitization campaigns and increased use of technology received lower scores despite being viewed positively. Participants explained that these interventions were considered less feasible due to financial constraints, limited technological infrastructure, and concerns regarding sustainability within rural community settings. This highlights the importance of contextual feasibility considerations during intervention development in low-resource environments.

Overall, the co-design findings demonstrate how iterative stakeholder engagement can facilitate the development of multidimensional intervention packages that respond to both the economic and social determinants of maternal and child health. The findings further reinforce the originality of the study by illustrating how existing community economic groups can be transformed into integrated platforms for reproductive and child health promotion through participatory co-design processes.

### 3.4 Findings from validation through insight gathering

During the validation interviews, participants expressed broad consensus that the proposed interventions reflected the lived realities and priority reproductive and child health needs of women participating in VICOBA groups and addressed important gaps within existing community structures (Table 4). Overall, the rough prototype intervention package was widely perceived as practical, culturally acceptable, and feasible to integrate within routine VICOBA activities. Participants particularly appreciated that the intervention package leveraged already existing community economic and social systems rather than introducing entirely new health-specific structures, which they believed would strengthen community ownership, acceptability, long-term sustainability and community ownership.

**Table 4:** Validation re-rating of the rough prototype interventions.

Rank	Intervention	Validation re-rating (/90)	Key validation insights
1	Maternal and child health education via VICOBA	88	Considered highly feasible because it could be integrated into existing VICOBA meetings with minimal additional costs.
2	Facilitate health insurance among VICOBA members	79	Viewed as highly relevant for reducing financial barriers to healthcare, although concerns regarding affordability remained.
3	Improve VICOBA governance/management	77	Participants emphasized the importance of strong leadership and transparent governance for sustainability of health-related activities.
4	Increase impact for vulnerable groups (AGYW/poor women)	76	Seen as socially valuable, but participants highlighted potential implementation challenges related to stigma, inclusion, and financial constraints.

However, the validation discussions also revealed important areas of critical reflection, disagreement, and implementation concern. Although the interventions received generally high ratings, participants did not uniformly endorse all components. Instead, discussions highlighted various perspectives regarding operational feasibility, affordability, leadership capacity, and sustainability within different VICOBA contexts. When participants re-rated the intervention components, the order of prioritization shifted slightly based on perceived practicality within existing VICOBA infrastructure, financial realities, group leadership dynamics, and membership composition.

Provision of MCH education through VICOBA meetings emerged as the highest-ranked intervention during validation. Participants explained that regular VICOBA meetings already bring women together consistently, making them convenient and cost-effective platforms for delivering structured health education. Women also perceived health education as highly achievable because it could be integrated into existing meeting schedules without requiring major financial investments or structural changes.

Facilitation of health insurance awareness and enrollment ranked second. Participants viewed this intervention as highly relevant due to the financial burden associated with maternal and child healthcare costs. Many women emphasized that improved understanding of insurance schemes and group-based facilitation mechanisms could reduce delays in healthcare seeking and improve preparedness during maternal emergencies. Nevertheless, some participants expressed concerns regarding affordability of insurance contributions among economically vulnerable households, suggesting that external partnerships and flexible contribution mechanisms would be necessary for successful implementation.

Strengthening VICOBA governance and management structures was also considered important for long-term sustainability of the intervention package. Participants noted that effective leadership, transparent financial management, and clear group constitutions would be essential for coordinating health-related activities and maintaining trust among members. However, a few participants raised concerns regarding variability in leadership capacity across different groups, which they believed could affect implementation consistency.

Similarly, increasing support for vulnerable women, including AGYW, was viewed positively but also generated important discussions regarding feasibility and inclusion. Participants acknowledged the social value of supporting younger and economically vulnerable women but noted that financial constraints, social stigma, and existing community perceptions could potentially limit participation of AGYW in some settings. Some participants also cautioned that poorly implemented targeting approaches could unintentionally create exclusion or social tension within groups. Consequently, participants emphasized that mentorship and inclusion strategies would need to be implemented carefully and sensitively to ensure equitable participation and community acceptance.

Most importantly, although validation ratings were generally high across all intervention components, the discussions revealed constructive concerns and differing opinions regarding sustainability, affordability, and operational feasibility. Inclusion of these perspectives strengthened the credibility of the validation process by demonstrating that participants critically reflected on both the strengths and potential limitations of the proposed interventions rather than uniformly endorsing all components.

Overall, the validation findings reinforced the relevance and contextual appropriateness of the co-designed intervention package while also providing important implementation insights that informed the final refinement and adaptation phase. The findings further demonstrate the value of iterative stakeholder engagement within HCD processes for strengthening intervention acceptability, feasibility, and responsiveness to community realities.

### 3.5. Findings from refinement and adaptation workshop

There was broad consensus among participants that the co-designed intervention package responded well to the current health, economic, and social realities faced by women participating in VICOBA groups. Experts agreed that the intervention aligned with growing interest in leveraging existing community economic structures as integrated platforms for strengthening women's resilience and improving maternal and child health outcomes in low-resource settings.

Participants emphasized that one of the major strengths of the intervention package was its ability to build upon already existing and socially accepted community systems rather than introducing parallel health structures. Experts noted that the regular organization of VICOBA meetings, established leadership systems, and strong social networks among members created favorable conditions for integrating reproductive and child health interventions into routine group activities. This was viewed as particularly important for enhancing community ownership, acceptability, and long-term sustainability of the proposed interventions.

Special attention during the workshop was given to operationalizing the highest-ranked intervention: provision of MCH education through VICOBA groups. Experts emphasized that while the concept was highly feasible and contextually appropriate, effective implementation would require a structured and standardized approach to health education delivery. Participants therefore recommended the development of a comprehensive training manual to guide facilitators, healthcare workers, and VICOBA leaders in delivering consistent and evidence-informed health education sessions during regular VICOBA meetings.

The proposed training manual was designed to address priority maternal and child health issues identified throughout the HCD phases while remaining practical and understandable for community members with varying levels of education and health literacy. Experts stressed that the manual should not only provide technical health information but also incorporate culturally sensitive communication approaches, participatory learning techniques, and practical guidance that reflects the lived realities of women within the study settings.

The manual includes key thematic areas related to maternal and child health, including nutrition during pregnancy, family planning, antenatal and postnatal care attendance, birth preparedness, recognition of danger signs during pregnancy and childbirth, vaccination, health insurance awareness, gender-based violence, and childcare practices. Participants agreed that inclusion of these topics would strengthen women's preparedness, improve health literacy, and enhance the ability of VICOBA groups to function as supportive platforms for maternal and child health resilience.

Importantly, experts also emphasized that implementation of the intervention package would require collaboration between VICOBA groups, community development officers, and healthcare providers to ensure technical support, supervision, and sustainability. Overall, the refinement findings demonstrate how iterative stakeholder engagement within the HCD framework contributed to the development of a contextually grounded and operationally feasible intervention package capable of integrating health promotion within existing community economic systems. The proposed topics for the training manual are presented as Table 5.

**Table 5:** Proposed topics for MCH training manual for VICOBA.

Module	Topics	Subtopics
Module 1: Nutrition for Pregnant Women	Food Groups (Protective Foods During Pregnancy)	Types of food groups; Nutrient-rich foods; Importance of protective foods during pregnancy
	Meal Frequency	Recommended number of meals during pregnancy; Balanced meal planning
	Benefits of a Balanced Diet	Maternal health benefits; Fetal growth and development
	Consequences of Poor Nutrition	Risks of inadequate diet; Maternal and fetal complications
Module 2: Family Planning	Importance and Benefits of Family Planning	Maternal health benefits; Birth spacing; Economic and social benefits
	Types of Family Planning Methods	Short-term methods; Long-term methods; Natural methods
	Challenges and Side Effects of Family Planning	Possible side effects; Addressing concerns
	Myths and Misconceptions About Family Planning	Common myths; Correct information and counseling
Module 3: Antenatal and Postnatal Clinic Attendance	Importance of Attending Clinics	Benefits of antenatal and postnatal visits
	Attendance During Pregnancy and After Delivery	Recommended number and timing of visits
	Family Involvement	Role of partners and family members in maternal care
	Key Tests During Clinic Visits	Essential maternal and fetal health screenings
Module 4: Danger Signs and Labor Signs	Danger Signs During Pregnancy	Symptoms requiring urgent medical attention
	Signs of Labor	Early and active labor indicators
	Duration of Labor	Expected labor stages and when to seek help
Module 5: Birth Preparedness	Delivery Supplies	Essential items for childbirth
	Place of Delivery	Importance of facility-based delivery
	Emergency Transport and Financial Preparedness	Planning for emergencies; Saving funds
	Birth Companion and Family Support	Role of companions during delivery
Module 6: Vaccination for Mother and Child	Health Insurance	Importance of maternal health insurance
	Benefits of Vaccination	Protection against diseases
	Types of Vaccines	Maternal and childhood vaccines
	Immunization Schedule	Recommended vaccination timeline
	Vaccine Side Effects and Challenges	Common reactions
	Managing Vaccine Challenges	Addressing concerns and side effects
Module 7: Rest, Exercise, and Work During Pregnancy	Importance of Adequate Rest	Benefits of rest for maternal and fetal health
	Risky Work During Pregnancy	Types of work that may harm mother or baby
	Safe Exercises During Pregnancy	Recommended physical activities
	Gender-Based Violence	Identifying and addressing violence during pregnancy
Module 8: Child Care After Birth	Growth Monitoring	Tracking child development and growth
	Babies with Low or High Birth Weight	Special care considerations
	Breastfeeding	Early initiation; exclusive breastfeeding
	Complementary Feeding	Appropriate feeding after six months

## 4 Discussion

This study applied an adapted HCD framework to co-design an intervention package aimed at strengthening women's resilience in addressing reproductive and child health (RCH/MCH) challenges through income-generating associations (IGAs), particularly VICOBA groups, in Tanzania. The findings demonstrate that women's economic groups represent important but underutilized community platforms for improving maternal and child health outcomes. Through iterative engagement with women, community leaders, and reproductive health stakeholders, the study identified a feasible and contextually grounded intervention package centered on four interrelated components: (i) maternal and child health education delivered through VICOBA meetings, (ii) facilitation of health insurance awareness and enrollment among members, (iii) strengthening governance and coordination within VICOBA groups, and (iv) increasing participation and support for vulnerable women, including AGYW. Collectively, these findings highlight the potential of integrating health promotion strategies into existing community economic structures as a sustainable approach for strengthening women's health resilience in low-resource settings.

Importantly, this study contributes to the growing body of literature emphasizing the importance of community-centered and participatory approaches in maternal and child health intervention development. Unlike many conventional top-down public health interventions that are externally designed and implemented with limited community ownership, the adapted HCD framework enabled continuous stakeholder engagement throughout the intervention development process. This iterative engagement ensured that proposed solutions reflected local priorities, implementation realities, sociocultural dynamics, and the lived experiences of women participating in IGAs. The findings therefore reinforce the argument that interventions developed through participatory co-design approaches may have greater contextual relevance, acceptability, and potential sustainability compared to externally imposed interventions.

### 4.1 IGAs as community resilience structures for reproductive and child health

The discovery inquiry findings revealed that VICOBA groups function not only as financial support systems but also as important social resilience structures that enable women to cope with household financial demands, respond to emergencies, and strengthen social solidarity. Women described these groups as critical safety nets that improve their ability to support children's education, stabilize livelihoods, and manage health-related financial crises. These findings align with broader evidence demonstrating that women's financial groups in low- and middle-income countries contribute not only to economic empowerment but also to enhanced social capital, collective efficacy, and community problem-solving capacity (22–24). Social networks formed through such groups may influence health behaviors by facilitating information exchange, emotional support, and collective action around shared community concerns.

Beyond financial empowerment, the findings suggest that IGAs may function as socially trusted community infrastructures capable of supporting broader public health objectives. Participants consistently emphasized that VICOBA groups already possess important implementation assets, including regular meetings, organized leadership systems, social trust, and strong interpersonal networks among women. These characteristics create favorable conditions for integrating reproductive and child health interventions into existing community structures without requiring establishment of parallel health-specific groups.

However, participants also emphasized that despite their strong organizational structures and social cohesion, VICOBA groups remain underutilized as platforms for reproductive and child health promotion. This finding highlights an important missed opportunity within existing community health systems. The World Health Organization has increasingly emphasized community engagement and community-owned structures as essential entry points for strengthening primary healthcare and health promotion interventions (14, 15). Leveraging existing women's economic groups for maternal and child health interventions may therefore provide a cost-effective, socially acceptable, and potentially sustainable alternative to establishing entirely new community health structures in resource-constrained settings.

The findings further suggest that integration of health promotion into IGAs may strengthen the interconnected relationship between women's economic resilience and health resilience. In many low-resource settings, financial vulnerability, gender inequality, and limited access to health information interact to shape maternal and child health outcomes. By embedding health interventions within community economic groups, the proposed intervention package attempts to address both structural and social determinants of health simultaneously.

### 4.2 Maternal and child health education through VICOBA as a priority intervention

Across all iterative HCD phases, participants consistently prioritized delivery of maternal and child health education through VICOBA meetings. During validation, this intervention emerged as the most feasible and acceptable component of the prototype package. Participants viewed routine VICOBA meetings as accessible and socially trusted spaces through which healthcare workers and trained facilitators could deliver essential information on antenatal care, nutrition, birth preparedness, recognition of danger signs during pregnancy, vaccination, newborn care, and health insurance.

These findings resonate with evidence from community women's group interventions implemented in several LMIC settings. A systematic review and meta-analysis by Prost et al. demonstrated that women's groups practicing Participatory Learning and Action (PLA) cycles contributed to reductions in maternal and neonatal mortality in settings with adequate participation and population coverage (30). Similarly, other studies have shown that participatory women's groups can strengthen maternal health knowledge, improve healthcare-seeking behaviors, and enhance collective problem-solving around maternal and newborn health challenges (19–23). However, it is important to acknowledge that the intervention proposed in the current study differs conceptually and operationally from PLA-based women's group approaches. Unlike PLA interventions, which are typically established specifically as health-focused participatory learning groups, the present intervention leverages already existing women's economic associations with established financial, social, and organizational functions. Rather than creating parallel maternal health learning structures, the intervention integrates reproductive and child health promotion within community economic groups that women already trust, attend regularly, and use for savings, mutual support, and livelihood activities. This distinction is particularly important for strengthening conceptual clarity and avoiding overgeneralization of findings from PLA-based interventions to the current context. The originality of the present study therefore lies not in establishing new participatory health groups, but in repositioning existing IGAs as integrated and potentially sustainable community platforms for reproductive and child health promotion. This approach may be especially relevant in low-resource settings where sustainability of externally established health groups can be difficult to maintain after project completion.

The refinement workshop further highlighted the importance of standardizing health education delivery through development of a structured maternal and child health training manual. Participants emphasized that implementation fidelity, scalability, and consistency of health messaging would require contextually appropriate educational materials tailored to the literacy levels, sociocultural realities, and daily experiences of women participating in VICOBA groups. These findings reinforce the findings in implementation science literature emphasizing the importance of structured delivery mechanisms and contextual adaptation in community-based interventions (19–23).

### 4.3 Health insurance facilitation as a strategy to address financial barriers

Another major contribution of the co-designed intervention package involved facilitation of health insurance education and enrollment among VICOBA members. Participants repeatedly described the financial burden associated with out-of-pocket healthcare payments and the challenges of accessing maternal and child health services without insurance coverage. Women viewed VICOBA groups as potentially effective platforms for improving awareness of available insurance schemes, facilitating group-based enrollment mechanisms, and supporting collective saving for healthcare needs.

These findings are particularly relevant within the Tanzanian context, where schemes such as the improved Community Health Fund (iCHF) were introduced to strengthen financial protection for households within the informal sector (31, 32). However, previous studies have shown that uptake of community health insurance schemes remain constrained by limited awareness, affordability challenges, and administrative barriers. The current findings therefore suggest that integrating insurance facilitation within community economic groups may simultaneously address informational, social, and financial barriers to healthcare access.

Importantly, the findings also reinforce broader arguments that maternal and child health outcomes cannot be separated from underlying economic conditions. Financial vulnerability often delays healthcare-seeking behavior, contributes to catastrophic health expenditures, and reduces women's ability to access timely maternal healthcare services. By integrating financial protection strategies into women's economic groups, the proposed intervention package seeks to strengthen preparedness for maternal and child health needs while simultaneously addressing broader social determinants of health.

### 4.4 Governance strengthening and inclusion of vulnerable women

Strengthening governance and coordination within VICOBA groups also emerged as an important intervention priority. Participants emphasized that transparent leadership structures, clear constitutions, and accountable financial management systems are essential for sustaining both economic and health-related group activities. These findings suggest that successful integration of health interventions into IGAs requires not only health education but also strengthening organizational capacity, collective accountability, and institutional trust within groups.

The co-designed package further emphasized increasing participation and support for vulnerable populations, particularly AGYW. Young women often experience disproportionate reproductive health risks alongside limited access to economic resources and social support systems (20, 21). Participants therefore proposed mentorship structures and inclusion mechanisms linking younger women with experienced VICOBA members to strengthen economic independence, health literacy, and collective support.

These findings align with broader global public health strategies emphasizing women's empowerment, social inclusion, and economic participation as pathways toward improving reproductive health outcomes. Importantly, the intervention package demonstrates how existing women-led community structures can potentially address multiple vulnerabilities simultaneously through integrated social, economic, and health-focused interventions.

### 4.5 Addressing gender dynamics and male engagement

The study further highlighted the significant influence of gender norms and household power dynamics on women's participation in IGAs and their ability to engage in reproductive and child health activities. Some women reported that male partners discouraged participation in VICOBA due to concerns regarding shifting household power relations, financial obligations, or changing social behaviors. Similar findings have been reported in other Tanzanian and sub-Saharan African studies where gender norms and male perceptions influenced women's participation in community programs and healthcare-seeking behaviors (6–8).

The findings suggest that while women-centered economic groups can strengthen empowerment and resilience, successful implementation of health interventions within these structures may require broader household and community engagement strategies. Male sensitization and inclusion strategies may therefore be important for reducing resistance, strengthening social support, and promoting shared responsibility for reproductive and child health outcomes. At the same time, the findings demonstrate the complexity of balancing women's empowerment initiatives within sociocultural environments where traditional gender norms continue to shape decision-making and participation in community activities.

### 4.6 Methodological contributions of the human-centered design approach

A key contribution of this study lies in its application of a HCD approach to maternal and child health intervention development within community-based income-generating associations. In this study, the HCD framework served as the overarching methodological orientation guiding the design, engagement, and refinement processes, while the HCD approach reflected the participatory and user-centered problem-solving philosophy underpinning the study. Grounded in participatory co-design principles described by Abookire et al., and related implementation science literature, the approach emphasized continuous engagement with end users and stakeholders to ensure that intervention ideas reflected local priorities, lived experiences, and contextual realities (25).

Unlike traditional top-down program development approaches, the HCD approach prioritizes collaborative learning, iterative engagement, and shared decision-making throughout the intervention development process. To operationalize this framework within the Tanzanian context, the study adapted the traditional HCD model into four iterative and interconnected phases: discovery and inquiry, consultative co-design and prototyping, validation and insight gathering, and refinement and adaptation (27). These iterative phases represented the operational steps through which stakeholders continuously contributed to identifying barriers, generating solutions, validating intervention priorities, and refining implementation strategies. The iterative nature of the approach enabled findings from one phase to directly inform subsequent phases, thereby strengthening contextual responsiveness and improving the relevance and acceptability of the proposed intervention package.

Importantly, the HCD approach allowed the research team to systematically integrate perspectives from women participating in IGAs, community development officers, IGA coordinators, and reproductive and child health experts throughout the study. This collaborative and iterative process enhanced the contextual grounding of the intervention package while also promoting stakeholder ownership, acceptability, and perceived feasibility within existing community structures. Although the study did not include real-world pilot testing of the prototype due to resource limitations, the validation phase provided important insights from women who were not involved in the initial co-design activities. This helped ensure that the proposed interventions reflected broader community perspectives and reduced the likelihood of overreliance on the views of the original co-design participants.

Future pilot implementation of the intervention package would require dedicated funding to support facilitator training, development and printing of educational materials, coordination with VICOBA leaders and healthcare providers, and implementation over an estimated period of 6–12 months. In addition, pilot testing would benefit from incorporation of a structured process evaluation to assess key implementation outcomes, including feasibility, acceptability, fidelity, participant engagement, and sustainability of the intervention package within routine VICOBA operations. Overall, the study demonstrates how an adapted HCD framework can be applied in low-resource settings to co-develop contextually appropriate, participatory, and potentially sustainable maternal and child health interventions embedded within existing community economic systems.

## 5 Strengths and limitations

This study has several strengths. First, the study engaged multiple stakeholders, including women in IGAs, community development officers, and reproductive health experts, ensuring that the intervention package incorporated diverse perspectives. Second, the use of the HCD framework enabled iterative refinement of the intervention components, enhancing their contextual relevance and potential sustainability. Third, the study was conducted in two regions with distinct sociocultural contexts, increasing the transferability of the findings.

However, several limitations should be acknowledged. The study relied primarily on qualitative data, and the intervention package has not yet been tested in a real-world implementation setting. Additionally, purposive recruitment through IGA coordinators may have introduced selection bias by including participants who were more actively engaged in group activities. Translation of interview transcripts from Swahili into English may also have resulted in minor loss of contextual nuance despite careful verification by the research team. Furthermore, the use of focus group discussions may have introduced social desirability bias, particularly during discussions related to sensitive issues such as gender norms, household dynamics, and male engagement in VICOBA activities. Although facilitators actively encouraged open participation, minimized power imbalances, and created supportive discussion environments, some participants may still have moderated their responses due to the group-based nature of the discussions.

## 6 Implications for future research and policy

The findings of this study suggest that integrating maternal and child health interventions into existing community economic groups may represent a promising strategy for strengthening community health systems. Future research should focus on pilot testing the co-designed intervention package to assess implementation outcomes such as feasibility, acceptability, and fidelity. Quantitative evaluations may also examine potential impacts on intermediate outcomes, including antenatal care attendance, birth preparedness, maternal health knowledge, and uptake of health insurance.

If demonstrated to be effective, this model could inform broader community-based health promotion strategies in Tanzania and other LMICs. Leveraging existing community economic groups may provide a scalable and sustainable pathway for strengthening women's resilience and improving maternal and child health outcomes in resource-constrained settings.

## Data Availability

The original contributions presented in the study are included in the article/Supplementary Material, further inquiries can be directed to the corresponding author.
